# Comparative study of Er^3+^-doped Ga-Ge-Sb-S thin films fabricated by sputtering and pulsed laser deposition

**DOI:** 10.1038/s41598-020-64092-3

**Published:** 2020-05-14

**Authors:** Simone Normani, Geoffrey Louvet, Emeline Baudet, Marek Bouška, Jan Gutwirth, Florent Starecki, Jean-Louis Doualan, Yannick Ledemi, Younes Messaddeq, Jean-Luc Adam, Petr Němec, Virginie Nazabal

**Affiliations:** 1 Department of Graphic Arts and Photophysics, Faculty of Chemical Technology, University of Pardubice, 53210 Pardubice, Czech Republic; 2 Univ Rennes, CNRS, ISCR (Institut des Sciences Chimiques de Rennes) – UMR 6226, F-35000 Rennes, France; 3 Centre d’Optique, Photonique et Laser (COPL), 2375 rue de la Terrasse, Université Laval, Québec, Qc Canada; 4 CIMAP UMR-CNRS 6252, Université de Caen Normandie, 14050 Caen, France

**Keywords:** Materials science, Materials for optics, Lasers, LEDs and light sources, Optical materials, Glasses

## Abstract

Despite the renewed interest in rare earth-doped chalcogenide glasses lying mainly in mid-infrared applications, a few comprehensive studies so far have presented the photoluminescence of amorphous chalcogenide films from visible to mid-infrared. This work reports the fabrication of luminescent quaternary sulfide thin films using radio-frequency sputtering and pulsed laser deposition, and the characterization of their chemical composition, morphology, structure, refractive index and Er^3+^ photoluminescence. The study of Er^3+ 4^I_13/2_ level lifetimes enables developing suitable deposition parameters; the dependency of composition, structural and spectroscopic properties on deposition parameters provides a way to tailor the RE-doped thin film properties. The surface roughness is very low for both deposition methods, ensuring reasonable propagation optical losses. The effects of annealing on the sulfide films spectroscopy and lifetimes were assessed. PLD appears consistent composition-wise, and largely independent of the deposition conditions, but radiofrequency magnetron sputtering seems to be more versatile, as one may tailor the film properties through deposition parameters manipulation. The luminescence via rare earth-doped chalcogenide waveguiding micro-structures might find easy-to-use applications concerning telecommunications or on-chip optical sensors for which luminescent sources or amplifiers operating at different wavelengths are required.

## Introduction

In recent times, the range of electromagnetic spectrum covering mid-wavelength infrared (MWIR) and long-wave infrared (LWIR), has become an appealing option for cutting-edge chemical sensing applications, due to its encompassing the excitation of fundamental vibrational transitions for many molecules, specifically in gaseous or in liquid phase^1^. Depicted interest in the mid-infrared range has raised the need for the development of novel low-loss optical devices, which in turn spurs the research also in the direction of new MWIR/LWIR radiation sources and amplifiers. As of now, the main light source operating in the infrared is the quantum cascade laser, which provides a wide emission wavelength range beyond the near-IR^2,3^ and therefore represents an important stepping stone in the advancements on this topic. Another crucial advancement is the miniaturization of sensors, which has been achieved by developing integrated optical components, allowing for compact and performant devices, and which is still a subject of research in order to optimize the means, the scale and the costs of the production.

Due to their low phonon energies and wide transmission window that extends far into the infrared region of the spectrum^4–6^, chalcogenide glasses doped with rare earths (RE) are essential to the development of integrated active optical devices in the near-wave infrared (NWIR)-to-LWIR range. Moreover, thanks to their high nonlinearities, chalcogenide glasses are particularly appealing for the development of all-optical signal processing^7–9^.

Amorphous chalcogenide thin films can be deposited relatively easily via radiofrequency (RF) sputtering^10–12^ and pulsed laser deposition (PLD)^13^, which makes it possible to obtain performant confined structures such as waveguides and resonators. In recent years, many advances have been made in the development of chalcogenide-based microstructures for *in situ* MWIR sensing, for instance in the medical field^14^ and for various environmental applications^15,16^. This field of research has the aim of paving the way for the development of easy, cheap and possibly large-scale production techniques^1^ for MWIR devices. Especially, erbium-doped quaternary chalcogenides such as Ga-Ge-Sb-S have been shown as a suitable choice for amplification and laser generation^17^ and for sensing applications in the MWIR. If one considers erbium for RE doping, some works have to be cited on various chalcogenide matrices like Ge_10_As_40_Se_25_S_25_:Er^3+^, 70Ga_2_S_3_:23La_2_S_3_:6La_2_O_3_:Er^3+^, As_2_S(Se)_3_:Er^3+^, Ge_33_As_12_Se_55_:Er^3+^, Ga-Ge-Se:Er^3+^ (Ga: 3–54 at. %, Ge: 0–35 at. %, Se: 21–70 at. %), Ga_5_Ge_20_Sb_10_S_65_:Er^3+^ for thin film deposition using RF sputtering method^17–21^, 0.15Ga_2_S_3_–0.85GeS_2_:Er^3+^, Ge_30_Ga_5_Se_64_Er_1_, Ga_5_Ge_20_Sb_10_S(Se)_65_, and GaLaS glass matrices deposited with PLD metod^22–25^, eventually (GeS_2_)_67_(Ga_2_S_3_)_33_:Er^3+^, As_2_S_3_:Er^3+^ or As_24_S_38_Se_38_:Er^3+^ matrices deposited by evaporation with erbium implantation and As_2_S(Se)_3_:Er^3+^ and Ge_3_Ga_28_Se_70_:Er^3+^ matrices by thermal co-evaporation^26–28^. Depending on the physical vapor deposition (PVD) method and the selected composition (with gallium or without, with arsenic or without, sulfides or selenides), the results obtained are relatively disparate and difficult to compare as the parameters change from one to another chalcogenide thin film. It should be noted that relatively few publications mention results within channelled guides allowing both a relatively efficient fluorescence in propagation mode coupled with limited optical losses and a concentration of erbium sufficiently high for integrated optics, and even fewer papers show luminescence in the mid-infrared. Under optical pumping at 1.55 µm, broadband guided mid-infrared photoluminescence was recorded for the first time above 3 µm from RE doped integrated chalcogenides waveguides^29^. Other rare earth-doped quaternary chalcogenide optical fibers with specific Ga-Ge-Sb-Se composition have also been successfully implemented in MWIR for gas sensing and for exploring the spectral range of LWIR using Tb^3+^, Dy^3+^ and Sm^3+^ ^30–32^. Given the properties of these amorphous materials, one can recognize the potential for applications of this kind of materials as both efficient sensors and laser sources, particularly in integrated optics. In this work, we show an extensive study of erbium-doped Ga-Ge-Sb-S thin films fabricated by PLD and RF sputtering, comparing the two PVD methods and the effects that the deposition conditions have on their composition, morphology, topography and optical properties. Erbium ion doping was chosen, although this RE is relatively ineffective in generating an emission in the mid-IR spectral range except around 2.7 μm, given a very unfavorable branching ratio to allow an emission at 4.5 μm. On the other hand, erbium allows us to follow its spectroscopic characteristics in the near infrared in sulfide thin films and thus to obtain very valuable information on the manufacturing process of rare earth-doped chalcogenide films devoted to infrared emission. Thanks to the knowledge of these different chemical, optical or spectroscopic characteristics put into perspective in relation to the deposition conditions, the aim of this study is to obtain a detailed understanding of the advantages of one PVD method over the other, and the possibilities of adapting the properties of the chalcogenide thin films by adjusting the set manufacturing parameters.

## Results and Discussion

In order to better control the manufacturing process of PLD and RF sputtered thin films of erbium-doped sulfides, as well as to better know the specific role and impact played by the two deposition techniques on the final results and characteristics, two sets of samples deposited by PLD and RF sputtering, were prepared and studied by recording their chemical composition, morphology, local structure and spectroscopy. The results of the analysis are presented in the following paragraphs, showing the dependence of various properties on the deposition parameters in the range of variation studied: in principle, we will show how the thin films resulting from the PLD technique are generally insensitive to variations in these parameter sets, whereas a trend can be identified for RF sputtering method.

### Chemical composition of sulfide thin films and targets

The chemical composition of the deposited films as well as the used targets was estimated via energy dispersive X-ray spectroscopy (EDS). The results are reported in Table 1, along with the refractive index estimation from variable angle spectroscopic ellipsometry (VASE).

**Table 1:** Experimental parameters of the for Er^3+^-doped Ga-Ge-Sb-S thin films deposited by PLD (Ar pressure, energy density (E.D.) and target-to-substrate distance (d)) and RF magnetron sputtering (Ar pressure, RF working power and distance), chemical composition of the films and its difference compared to the used targets (Δ%Ga, Δ%Ge, Δ%Sb, Δ%S) and refractive index difference (at 1.55 µm) between films and targets. Note that the chemical composition and the refractive index of the targets are Ga_5_Ge_19_Sb_10_S_66_ with *n*_1.55 µm_ = 2.24 for PLD and Ga_4_Ge_19_Sb_9_S_68_ with *n*_1.55 µm_ = 2.25 for sputtering.

Sample	PLD Samples								
	*p*	E.D.	*d*	Chemical Composition (±1 at.%)					Ref. Index
	mbar	J/cm^2^	cm	Real composition	Δ%Ga	Δ%Ge	Δ%Sb	Δ%S	Δ*n* (±0.01) 1.55 µm
**P-1**	N/A	2.4	6	Ga_8_Ge_23_Sb_14_S_55_	2	5	4	−11	0.26
**P-2**	N/A	2.1	6	Ga_7_Ge_23_Sb_13_S_57_	2	4	3	−9	0.19
**P-3**	N/A	1.7	6	Ga_7_Ge_22_Sb_14_S_57_	2	4	3	−9	0.22
**P-4**	N/A	2.4	8.5	Ga_8_Ge_23_Sb_12_S_57_	2	5	2	−9	0.19
**P-5**	5·10^−4^	2.4	6	Ga_8_Ge_23_Sb_14_S_55_	2	4	4	−10	0.21
**P-6**	5·10^−3^	2.4	6	Ga_8_Ge_23_Sb_14_S_55_	2	5	4	−11	0.26
**P-7**	5·10^−3^	2.1	6	Ga_8_Ge_23_Sb_14_S_55_	2	5	4	−11	0.25
**P-8**	5·10^−3^	1.7	6	Ga_8_Ge_23_Sb_13_S_56_	2	4	3	−9	0.20
**P-9**	5·10^−3^	2.4	8.5	Ga_8_Ge_23_Sb_14_S_55_	3	5	3	−11	0.29
**P-10**	5·10^−2^	2.4	6	Ga_7_Ge_23_Sb_14_S_56_	2	4	4	−10	0.25
**P-11**	N/A	2.4	7.25	Ga_8_Ge_24_Sb_13_S_55_	3	5	3	−11	0.36
**P-12**	5·10^−3^	2.4	7.25	Ga_8_Ge_24_Sb_15_S_53_	3	6	4	−13	0.31
**Sample**	**Sputtered Samples**								
	***p***	**RF power**	***d***	**Chemical Composition (±1 at.%)**					**Ref. Index**
	**mbar**	**W**	**cm**	**Real composition**	**Δ%Ga**	**Δ%Ge**	**Δ%Sb**	**Δ%S**	**Δ*n*** **(±0.01) 1.55 µm**
**S-1**	7·10^−3^	20	5	Ga_8_Ge_24_Sb_12_S_56_	4	5	2	−11	0.13
**S-2**	7·10^−3^	15	5	Ga_8_Ge_24_Sb_12_S_56_	4	5	3	−12	0.20
**S-3**	7·10^−3^	10	5	Ga_8_Ge_24_Sb_13_S_55_	4	5	4	−13	0.15
**S-4**	7·10^−3^	20	8.5	Ga_7_Ge_23_Sb_11_S_59_	3	4	2	−9	0.07
**S-5**	8.5·10^−3^	20	5	Ga_7_Ge_24_Sb_11_S_58_	3	4	2	−9	0.11
**S-6**	8.5·10^−3^	15	5	Ga_7_Ge_23_Sb_12_S_58_	3	4	3	−10	0.11
**S-7**	1.10^−2^	25	5	Ga_7_Ge_23_Sb_12_S_58_	3	4	2	−9	0.09
**S-8**	1·10^−2^	20	5	Ga_7_Ge_24_Sb_11_S_58_	3	5	2	−10	0.09
**S-9**	1·10^−2^	15	5	Ga_7_Ge_24_Sb_11_S_58_	3	4	2	−9	0.09
**S-10**	1·10^−2^	10	5	Ga_7_Ge_23_Sb_12_S_58_	3	4	3	−10	0.10
**S-11**	1·10^−2^	20	8.5	Ga_7_Ge_22_Sb_12_S_59_	3	3	2	−8	0.13
**S-12**	2·10^−2^	20	5	Ga_7_Ge_24_Sb_12_S_57_	4	5	2	−11	0.10
**S-13**	2·10^−2^	15	5	Ga_6_Ge_21_Sb_13_S_60_	3	2	3	−8	0.05
**S-14**	3·10^−2^	20	5	Ga_7_Ge_23_Sb_11_S_59_	3	4	2	−9	0.02
**S-15**	3·10^−2^	15	5	Ga_6_Ge_22_Sb_12_S_60_	3	3	2	−8	0.01
**S-16**	7·10^−3^	20	6.75	Ga_8_Ge_23_Sb_10_S_59_	4	4	1	−9	0.03
**S-17**	1·10^−2^	20	6.75	Ga_7_Ge_24_Sb_11_S_58_	4	4	2	−10	0.01

One can see that the deposited films all share a deficiency in sulfur concentration with respect to the target composition, which is a little bit more pronounced in the PLD samples (an average of 10 at. % vs. 9 at. % for the sputtered films). Consequently, the other elements concentration is on average higher than the target’s, and in particular, the Ge and Sb concentration is sensibly larger in the PLD thin films than in the bulk, as is the Ge and Ga concentration in RF-sputtered samples. This can be understood in the case of sputtering method by difference in sputtering rate of sulfur and antimony compared to gallium and more over germanium atoms^12,33^. Whatever the PVD technique used, chalcogen atoms are always deficient in composition. Less energetic, lighter, they must probably be more sensitive to the dynamic vacuum created in the chamber, to the renewal of the process gas, and consequently less deposited on the substrate than the other atoms. It should also be noted that this chalcogen deficit will be more or less marked from one deposition chamber to another and will depend mainly on the volume of the chamber, the position of the targets in relation to the secondary vacuum pump, the gas flow in the chamber and its aeraulic.

**Figure 1:**
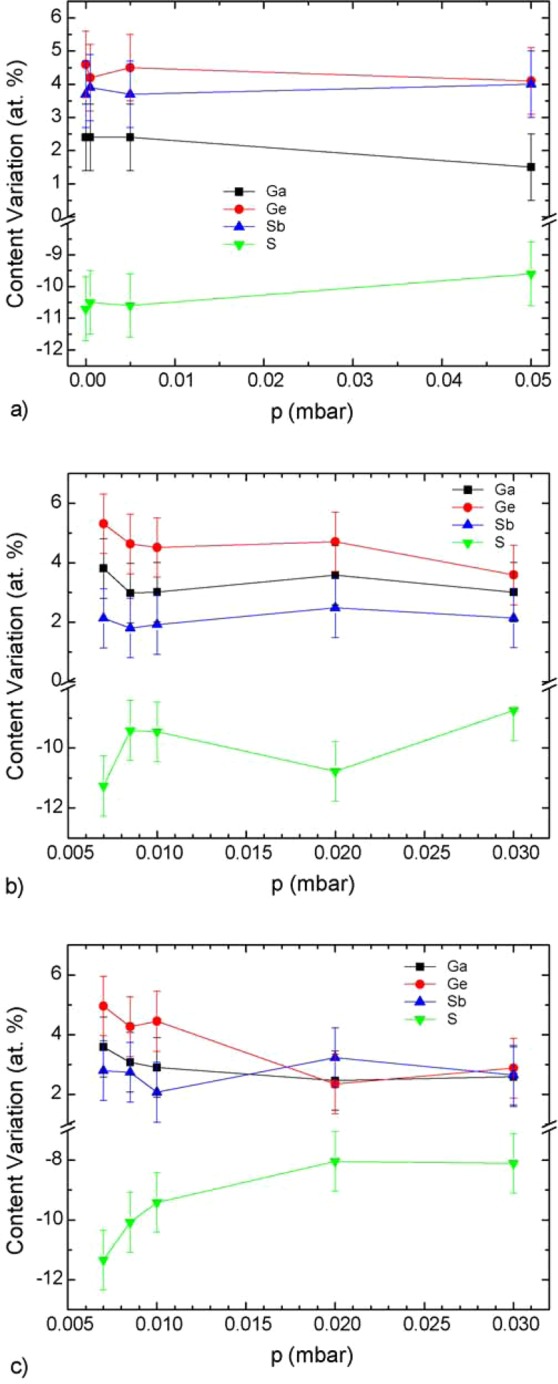
Plot of the variation in Ga, Ge, Sb, S concentration as a function of pressure during deposition for PLD Ga_5_Ge_20_Sb_10_S_65_:Er^3+^ samples (**a**) and sputtered samples deposited at 20 W (**b**) and 15 W (**c**).

The chemical elements concentration was plotted as a function of the deposition parameters. The trends were then observed as a function of chamber pressure. In fact, one can see that while the sulfur deficiency is for the most part independent of the argon pressure in the case of PLD films (Fig. 1a), it follows a decreasing trend as a function of pressure in sputtered thin films whatever the RF power as it was already observed in the case of Ge-Sb-Se sputtered films^12,34^ (Fig. 1b,c).

### Morphology, topography and refractive index of Er^3+^-doped Ga-Ge-Sb-S thin films

**Figure 2:**
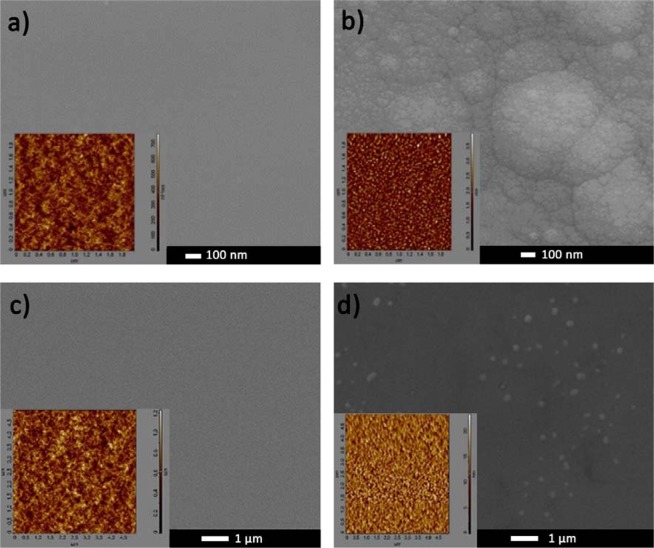
SEM and AFM images of as-deposited PLD thin films a) P-6 (5·10^−3^ mbar, 2.4 J/cm^2^, 6 cm), (**b**) P-10 (5·10^−2^ mbar, 2.4 J/cm^2^, 6 cm), and sputtered thin films (**c**) S-7 (1·10^−2^ mbar, 25 W, 5 cm), (**d**) S-14 (3·10^−2^ mbar, 20 W, 5 cm).

The thin films thickness and refractive index at the telecommunication wavelength were determined by the analysis of VASE data. The deposition rate of PLD thin films (ranging from 18 to 63 nm/min) is consistently higher than that of sputtered samples (ranging from 3 to 18 nm/min), which is typical for PLD when compared to the RF-sputtering process of dielectric targets, even enhanced by magnetron. From the AFM and SEM measurements it appears that the morphology of the samples is not strongly influenced by power, energy density, nor target-to-substrate distance, and most samples show quite low root mean square surface roughness (below 0.5 ± 0.1 nm, scanned area of 5 µm × 5 µm). In addition, sputtered samples appear to have the better surface morphology, with generally somewhat lower roughness than PLD samples (under 0.3 ± 0.1 nm). However, as one can see in Fig. 2, samples deposited with relatively high chamber pressure have very high rough surfaces (up to more than 4 nm), which is in accordance with expectations, as the gas provides additional resistance to the extracted particles flow, therefore the deposited film homogeneity.

**Figure 3:**
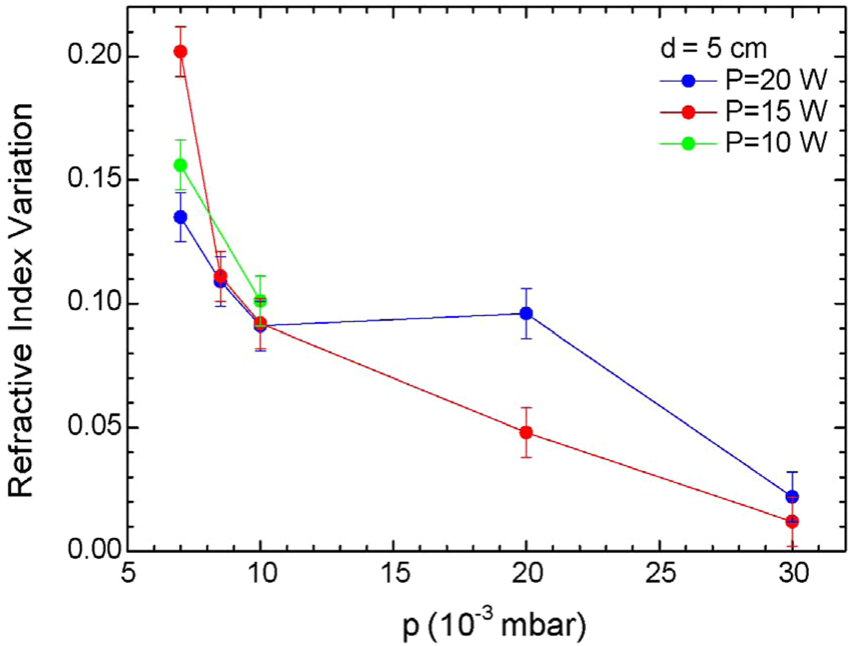
Plot of refractive index variation (deviation from bulk value *n* = 2.249) between thin films and Er^3+^-doped Ga-Ge-Sb-S target as a function of Ar pressure for sputtered samples.

The refractive index difference between the films and the targets was investigated as a function of the different deposition parameters. One can see from Table 1 that the films’ refractive index is always greater than that of the bulk targets. While most parameters (energy density, RF power, target-to-substrate distance) do not show any univocal effect on refractive index, it was observed that pressure seems to play a definite role. PLD samples do not show a change in the refractive index that would follow a pronounced behavior. The refractive index of sputtered samples, instead, appears to be influenced by pressure. Figure 3 shows the variation of the sputtered thin film refractive index as a function of argon pressure in the chamber: the mismatch decreases with increasing argon pressure, and it is a consistent behavior across all deposition powers considered. This relationship is interesting as it suggests that it is possible to tailor the refractive index of RF sputtered thin films, opening the way for the sputtering fabrication of multilayer and graded-index devices with the use of a single glass target, by adjusting the argon pressure in the chamber. The observation of a dominant role of pressure on the thin films composition in the case of RF sputtering is in agreement with previous results obtained in the study of Ge-Sb-Se films as reported by Baudet *et al*.^12^.

**Figure 4:**
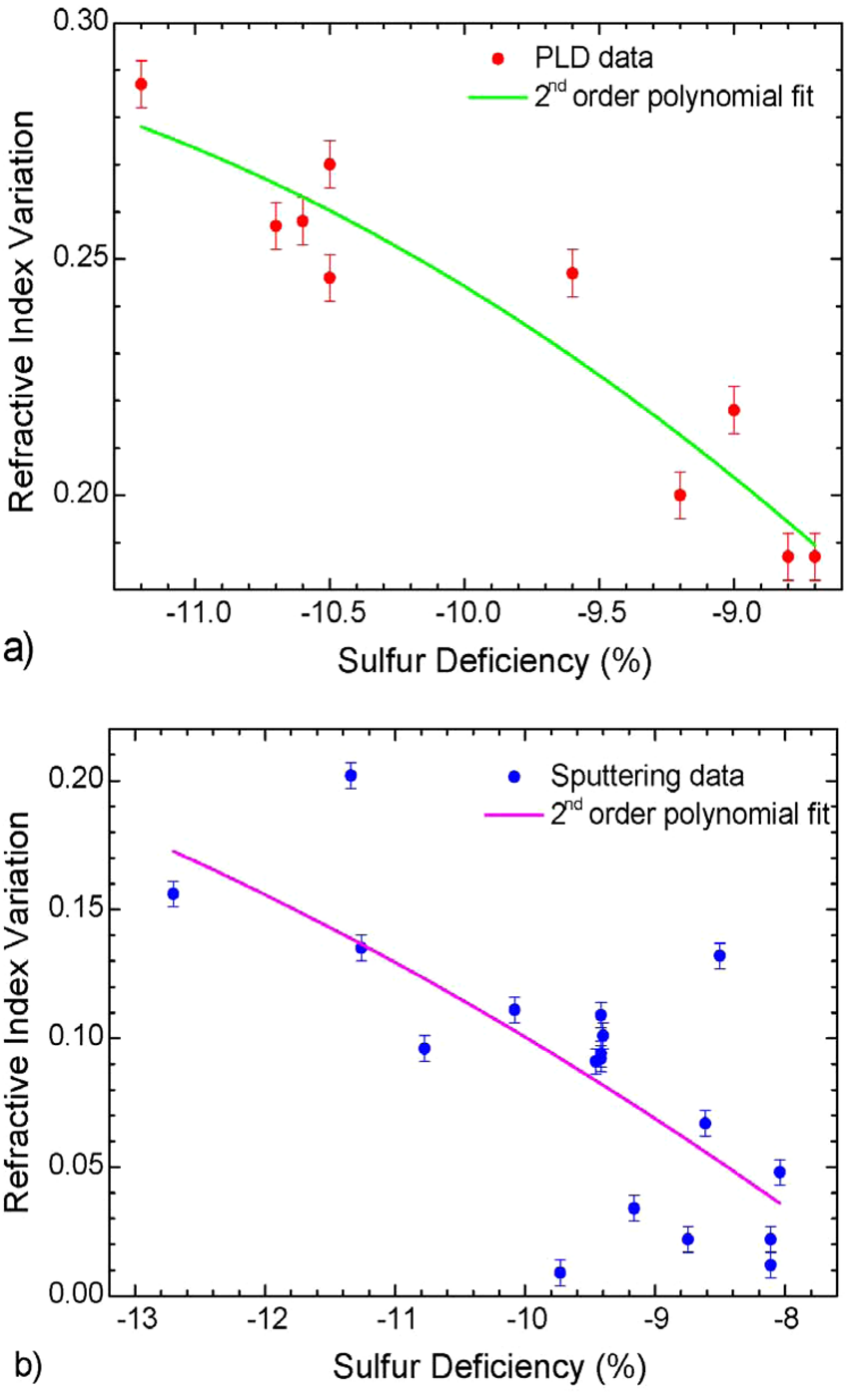
Plot of refractive index variation between thin film and Er^3+^-doped Ga-Ge-Sb-S target as a function of the variation in sulfur concentration, for (**a**) PLD samples and (**b**) sputtered samples. The polynomial fit shows the approximate data trend.

By plotting the refractive index mismatch as a function of the sulfur concentration one can observe a decreasing trend (Fig. 4). The trend is better defined in PLD samples, while in sputtered films the data are more scattered, although the general decreasing behavior is still visible and the overall range of this fluctuation is larger, due to the greater change in sulfur concentration discussed previously.

### Raman scattering spectroscopy of Er^3+^ sulfide thin films

**Figure 5:**
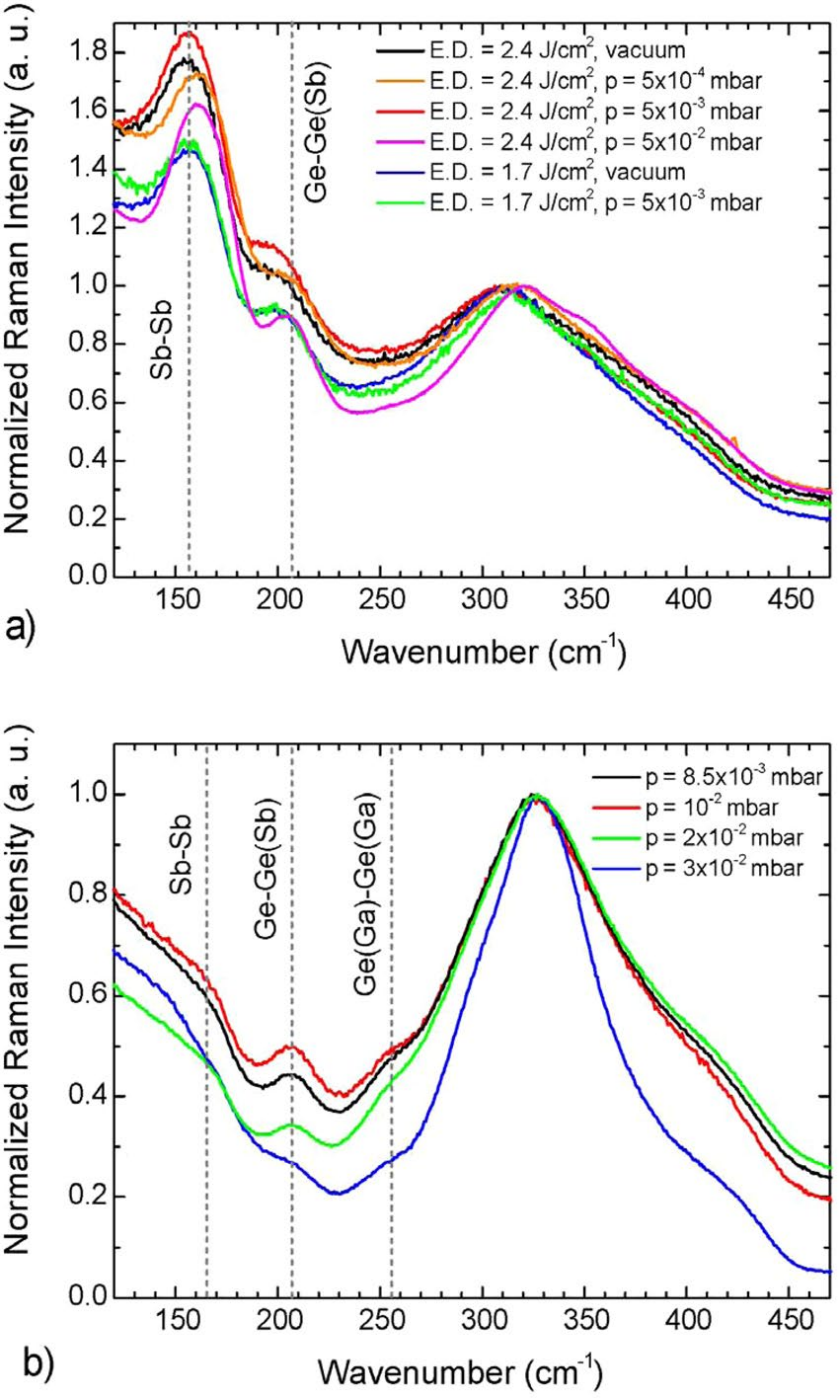
Raman scattering spectra of as-deposited Er^3+^-doped Ga-Ge-Sb-S thin films prepared by (**a**) PLD (target-to-substrate distance 6 cm) and (**b**) RF magnetron sputtering (power 20 W, target-to-substrate distance 5 cm).

Raman scattering spectroscopy was employed to investigate the thin film local structure. Figure 5 shows the Raman scattering spectra of various films deposited by PLD and RF-sputtering under similar condition (i.e. laser energy density, RF power and target-to-substrate distance) and varying in-chamber argon pressure. The main Raman feature of sulfide films is located between 300 and 450 cm^−1^: it should be expected to observe the symmetric stretching modes of [SbS_3/2_] pyramidal units, [GaS_4/2_] and [GeS_4/2_] tetrahedra, detectable around 296, 320, and 340 cm^−1^. Shoulder at 370 cm^−1^ is connected with companion mode (A_1_^c^), linked to the vibrations of [GeS_4/2_] tetrahedra bound by their edges. The other shoulder above 400 cm^−1^ can be associated mainly with the S_3_Ge–S–GeS_3_ vibrations related to edge sharing or corner sharing of [GeS_4/2_] tetrahedra^24^. Nevertheless, they cannot be distinctly detected due to the overlap with the Er^3+^ fluorescence under laser probe excitation at 785 nm or 633 nm. The Raman bands and shoulders clearly present at 155, 205, and 255 cm^−1^ were identified as markers of the presence of Sb-Sb, Ge-Ge(Sb), and Ge(Ga)-Ge(Ga) bonds respectively usually not present for a stoichiometric composition of the bulk glass and often associated in literature to defects or “wrong” bonds. One can observe that no significant effect is clearly visible in the case of PLD when changing the deposition parameters, while the presence of these specific bonds decreases with increasing Ar pressure in the RF sputtered samples, as seen by the gradual reduction in the intensity of the associated Raman features. One can also notice strong differences between the PLD and sputtered samples “wrong” bond ratios. The Ge(Ga)-Ge(Ga) bonds are more prominent in sputtered samples and more difficult to observe in PLD films because of the widening of the main band around 300 cm^−1^ generally associated with a larger disorder and also a slightly higher concentration of antimony in PLD films. Overall, “wrong” bonds seem to be in greater proportion in PLD films, especially Sb-Sb bond vibration is more intense in PLD films than sputtered ones (Fig. 5). This is consistent with the larger content of antimony and deficit in sulfur in the PLD films (Fig. 1 and Table 1) and is the signature of singular cluster favoring Sb-Sb bonds for PLD films compared to sputtered films, probably related to the different nature of the plasma generated by these two deposition methods.

### Er^3+^ spectroscopic characterization

**Figure 6:**
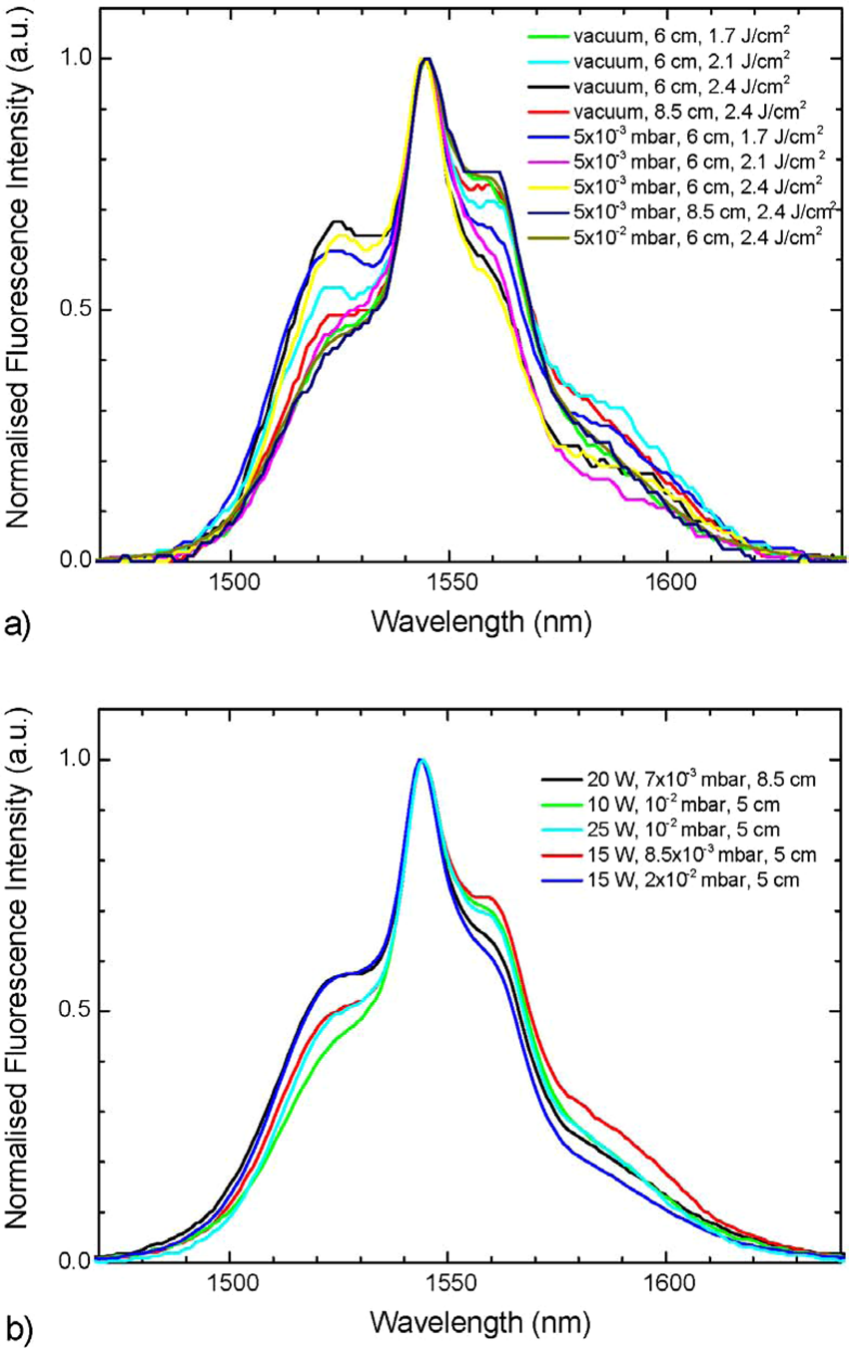
Photoluminescence spectra of Er^3+^-doped Ga-Ge-Sb-S thin films prepared by (**a**) PLD and (**b**) RF magnetron sputtering, collected exciting at 980 nm.

The 1.55 µm fluorescence spectra of the pristine samples (Fig. 6) show that there is a significant difference between the sputtered and PLD films. Noteworthy, while there is variation in overall intensity from one sample to the other one, the shape of the fluorescence band for sputtered films remains roughly the same. On the other hand, PLD samples display much greater variations in the relative intensity of the peaks that make up the spectral band, particularly the shoulders at ~1520 and 1560 nm. In principle, this may be linked either to reabsorption effects from the erbium ions within the layer which is more impactful at shorter wavelengths, or to a higher degree of a local structural disorder (which could be considered based on Raman spectra analysis) of the PLD films that distorts the local crystal field at the dopant sites, changing the Stark levels ratios, and thus the emission band shape. The presence of more defects and greater inhomogeneity may be correlated with the somewhat larger surface roughness observed previously by AFM with respect to the sputtered samples. Moreover, since the measurements were taken with an incident-light configuration on micrometer-thick films, reabsorption is expected to affect the emission band shape to a much lesser degree than in the case of a bulk or waveguide sample. The normalization at the peak maximum was therefore chosen over the conventional one at the longer wavelength features less affected by reabsorption, in order to visually enhance the band shape variations, and illustrate how the extent of such fluctuations is greater for the PLD films than the sputtered ones.

**Figure 7:**
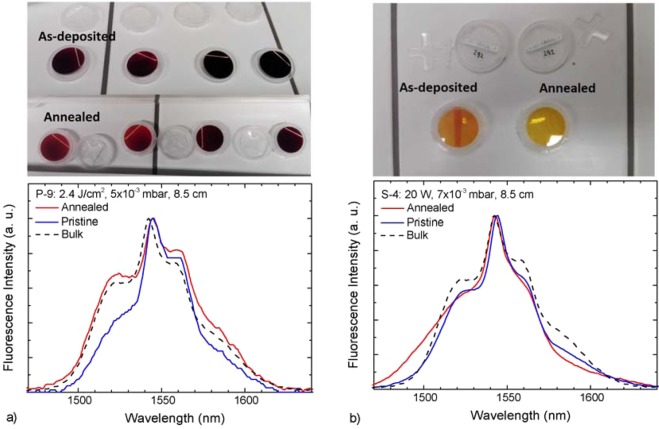
Comparison of fluorescence spectra before and after annealing for (**a**) PLD films and (**b**) sputtered films and photographs of PLD (left) and sputtered (right) thin films (1″ samples) after and before annealing.

The effects of annealing on the fluorescence spectra can be observed directly in Fig. 7. One can notice that the changes in spectral band shape are more pronounced in the case of PLD samples. This phenomenon suggests that the fluctuations previously observed in as-deposited PLD films spectra are due to the inhomogeneity of the local lattice environment around the Er^3+^ ion positions, rather than an effect of fluorescence reabsorption, as well as of the greater degree of film stress removal by annealing. Several samples (both PLD and sputtered) show a tendency to increase the intensity ratio for the short-wavelength emission contributions compared to the longer-wavelength ones: this could signify a reduction in the aforementioned self-absorption after the annealing, which is potentially associated to the band-gap energy variation with the annealing. However, since it is not a change that takes place consistently across the samples, one cannot ascribe the shape variation only to this phenomenon. From the comparison with the bulk emission band at 1.55 µm, it can also be noticed that PLD films have a more similar emission band shape to that of bulk glass, which in turn suggests a more similar local amorphous structure in the vicinity of erbium centers, and are more clearly affected by reabsorption than sputtered films. On the contrary, the sputtered samples display a smoother, narrower emission band shape.

The 980 nm emission was also collected for various samples. The shape of the emission band is rather stable across samples, and the intensity seems to vary depending on the deposition parameters similarly to the 1.55 µm emission. The observation of emission peak around 1060 nm, which is not consistent with regular Er^3+^ fluorescence, suggests the presence of neodymium contaminants in the glass substrate, which contributes to hinder the erbium emission and was observed in previous works.

**Figure 8:**
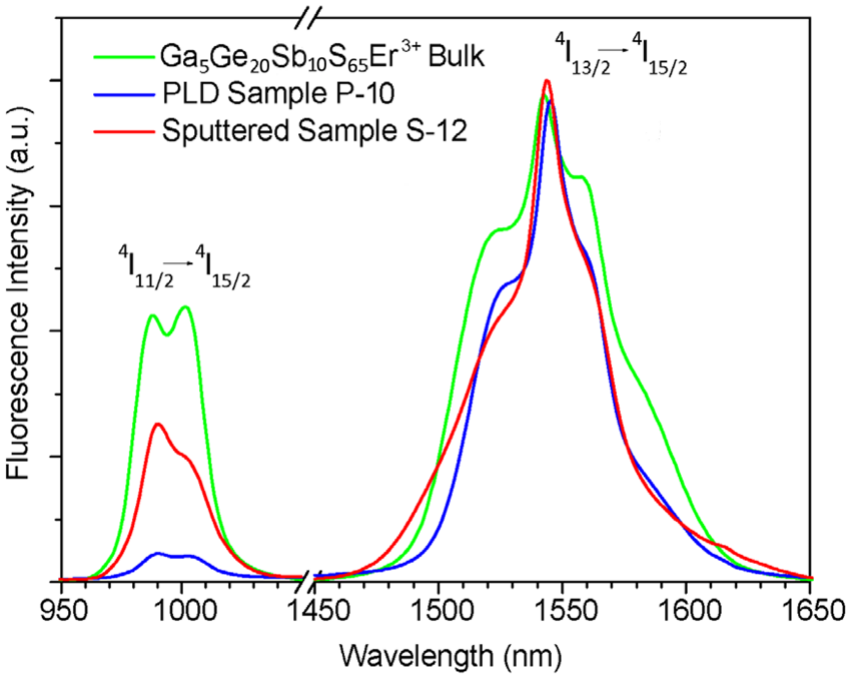
Emission spectra under 808 nm excitation, showing the ratio between the ^4^I_11/2_→^4^I_15/2_ and the ^4^I_13/2_→^4^I_15/2_ transitions for a glass bulk, a PLD sample, and a sputtered sample (both annealed).

Fluorescence spectra under 808 nm excitation were collected to include both the ^4^I_11/2_→^4^I_15/2_ and ^4^I_13/2_→^4^I_15/2_ transitions in order to observe the variation of the intensity ratio between the two emission bands. The measurement was performed on a bulk sample (a fragment of the sputtering target, Fig. 8 - green line), and the samples with highest overall fluorescence intensity, one PLD (P-10, Fig. 8 - blue line) and one sputtered (S-12, Fig. 8 - red line). The first observation is that the 1550 versus 980 nm emission intensity ratio is quite different from that of previous works on similar materials (Moizan *et al*.^35^), where the ratio for Ga-Ge-Sb-S bulk glass with 1000 ppm Er^3+^ doping is in favor of the 980 nm peak, while in our measurements the latter is sensibly lower than the 1550 nm one. This difference could be attributed to a more pronounced effect of energy transfer and cross-relaxation from the ^4^I_11/2_ level in our samples, due to the higher Er^3+^ concentration. Moreover, it was thought that a portion of the material in deposited thin films may be crystalline, which would further contribute to favor the energy transfer processes due to the -in average- higher host matrix crystal field. However, no clear suggestion of crystallite formation was visible from spectroscopy measurements, as no structuring of the emission peaks indicates the presence of crystalline phase, which is in agreement with observations in previous works^24^, suggesting that the change in the aforementioned emission ratio is purely an effect of optical quenching due to the high dopant concentration. In addition, the measurements show that the intensity ratio shifts further in favor of the ^4^I_13/2_→^4^I_15/2_ emission in deposited thin films, and in fact, the effect is more pronounced in PLD samples. This phenomenon is attributed to the presence of structural defects in the material, especially in the proximity of active ion sites, which greatly increase the probability of non-radiative relaxation processes, and therefore correspondingly reduce the ^4^I_11/2_ population. This is in agreement with the observation of PLD samples being in general slightly rougher, presenting higher proportion of “wrong” bonds or defects than sputtered samples (suggesting a somewhat greater degree of structural inhomogeneity).

The luminescence in the MWIR range was assessed for bulk sputtered target, PLD and sputtered films by exciting at 808 nm and collecting the emission around 2.7 µm, corresponding to the ^4^I_11/2_→^4^I_13/2_ emission. Although in several films, it was possible to observe the expected emission band, the signal-to-noise ratio was very low due to the non-propagating geometry used for the standard spectroscopy measurements. The objective of this study was not to obtain a high-intensity MWIR emission that would require photolithography and waveguide etching, but simply to check whether thin layers with the highest intensity at 1.5 µm can actually emit around 2.7 µm.

**Figure 9:**
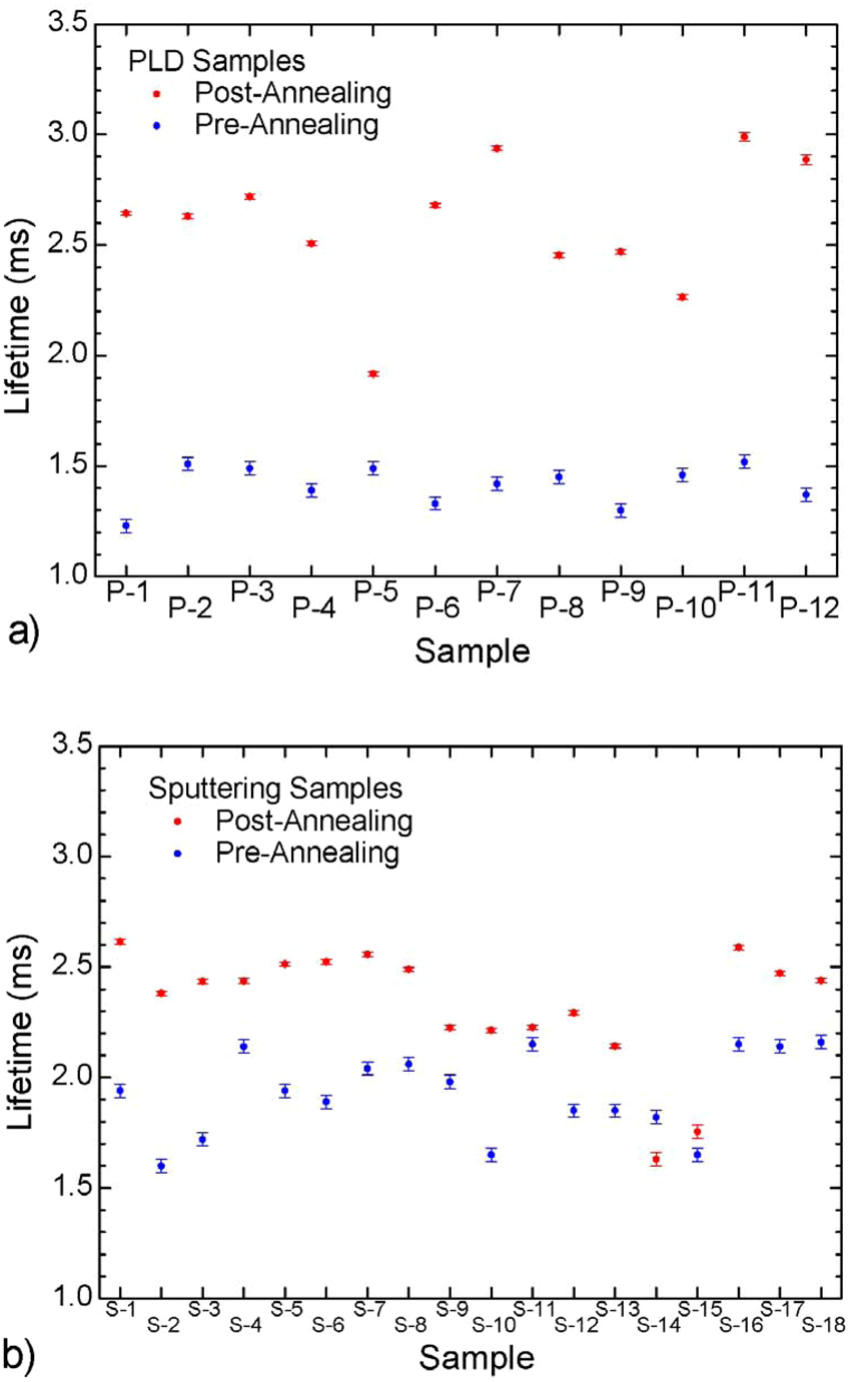
1.55 µm fluorescence lifetimes of PLD and sputtered films before and after the annealing for (**a**) PLD films and (**b**) sputtered films.

The lifetimes of 1.55 µm fluorescence were obtained fitting the time-resolved emission intensity data with single-exponential decay functions. The resulting lifetime values before and after annealing were compared. One can clearly assess (Fig. 9) that the annealing process causes an increase in fluorescence lifetime both for sputtered and PLD samples, but much more accentuated for the latter. In fact, the lifetimes of PLD samples, which were sensibly lower than those of the sputtered films before annealing, are now very similar to them. This fact could also support the claim that the annealing has a greater effect on PLD samples due to the larger number of structural defects within the latter, in as-deposited state.

**Figure 10:**
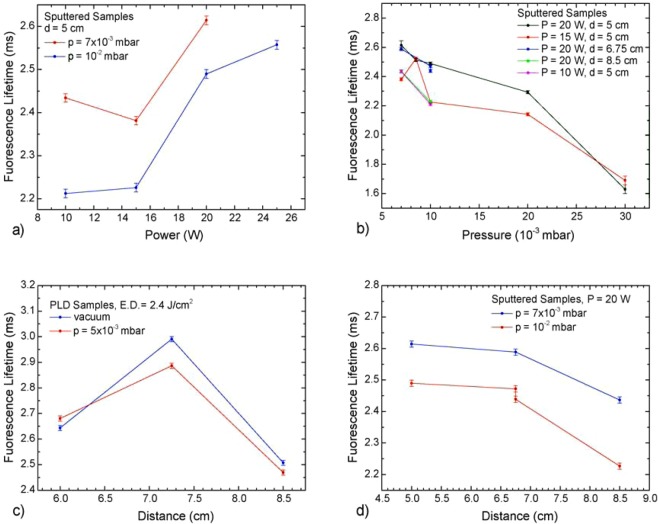
Plot of fluorescence lifetimes versus the different deposition parameters for sputtered samples (**a,b,d**) and for PLD versus target-to-substrate distance (**c**). The lines between data points are a guide for the eye.

With these data at hand, one can examine the dependence of both fluorescence intensity and fluorescence lifetime on the deposition parameters. Figure 10 shows the behavior of fluorescence lifetimes as functions of deposition pressure, target-to-substrate distance, and RF power. While it is not possible to extrapolate a well-defined trend of fluorescence intensity as a function of laser energy density, RF power, target-to-substrate distance and argon pressure in the chamber, one can deduct from Fig. 10 some definite behaviors. In particular, while the lifetime of PLD samples cannot be clearly described as a function of either energy density or Ar pressure, one can identify the dependency of the sputtered samples lifetimes as a function of power and pressure: the sputtered films lifetimes tend to increase along with the power applied to the cathode, and decrease with increasing Ar pressure. Moreover, the lifetime of sputtered samples follows a decreasing trend as a function of target-to-substrate distance; however, the same does not apply to PLD samples (Fig. 10c), where it seems the lifetime has a maximum within the range considered, as it is highest for the samples deposited at 7.25 cm.

In general, we can also remark that PLD samples have a much less clear dependency on the deposition parameters, as though their properties do vary with them, they do not have a univocal relationship to pressure and energy density. On the other hand, the sputtered samples plots suggest an increase in the fluorescence lifetime with increasing applied power during deposition (Fig. 10a), and a decrease with increasing Ar pressure (Fig. 10b), while a straightforward behavior of fluorescence intensity as a function of laser energy density, power, or Ar pressure is almost impossible to define. It is worthwhile to point out that the lifetimes and fluorescence intensities appear to follow opposite trends in the case of PLD as function of pressure and distance, which is consistent with the general expectation of lifetime and intensity being inversely proportional (compare Fig. 10c,d), while this relation is not as evident in the other cases. It is thought that the lack of this proportionality be due to the strong presence of the non-radiative relaxation processes from the ^4^I_13/2_ level, possibly rising from the presence of the structural defects, as observed when exciting at 808 nm.

## Methods

Chalcogenide glasses with the intent of obtaining a nominal composition of Ga_5_Ge_20_Sb_10_S_65_ and doped with Er^3+^ (0.5 wt.%) were prepared by means of conventional melt and quenching method. Glasses were synthesized from high purity (5 N) commercial elements^17,24,35^. Sulfur was further pre-purified by a static distillation. Elements were weighted in a dry gloves box in appropriate amounts and introduced in a silica ampoule under vacuum for few hours. Then, the ampoule was sealed and elements were heated in a rocking furnace at 900 °C during 12 hours to ensure the homogenization of the melt. After the quenching in water, the glass was annealed below the glass transition temperature (T_g_ ≈ 305 °C) for 3 hours. Targets were obtained after slicing and polishing the glass rods. Chalcogenide targets were prepared in the form of glass cylinders, 3 mm thick with a 25 and 50 mm diameter for PLD and RF magnetron sputtering deposition respectively^17,24^. The actual compositions were Ga_5_Ge_19_Sb_10_S_66_ and Ga_4_Ge_19_Sb_9_S_68_, for the PLD and sputtering targets respectively.

Er^3+^-doped Ga-Ge-Sb-S thin films were obtained by PLD (named from P-1 to P-12) and by RF magnetron sputtering (named from S-1 to S-18) (Table 1)^17,24^. In the case of PLD deposition, chalcogenide targets were ablated with a KrF excimer laser emitting at 248 nm using 160, 120 and 85 (±3) mJ output pulse energies, pulses duration of 30 ns and 20 Hz repetition rate. Laser energy densities were set at 2.4, 2.1 and 1.7 J/cm^2^ depending on pulse energy. Various Ar pressures were used during deposition: 5·10^−2^ (Ar flow 75 sccm), 5·10^−3^ (Ar flow 25 sccm), 5·10^−4^ mbar (Ar flow 15 sccm) and no Ar gas (vacuum about 2·10^−7^ mbar). In order to obtain uniform film thickness, the off-axis PLD technique combining rotating substrates and targets was used. Rotation speeds were about 15 and 30°/sec for targets and substrates respectively. Substrates were positioned parallel to the target surface at a distance of 6, 7.25, and 8.5 cm. In the case of RF magnetron sputtering, considering the insulator character of the chalcogenide target, sputtered films were deposited at low RF working power: from 10 to 25 W. In order to study the influence of Ar pressure, five Ar working pressures were applied for the sputtering process: 7·10^−3^, 8.5·10^−3^, 1·10^−2^, 2·10^−2^ and 3·10^−2^ mbar (Ar flow was set at 75 sccm for all sputtering depositions). An off-axis substrate rotation (30°/sec) was used during deposition while substrates were positioned at a target-to-substrate distance of 5, 6.75 and 8.5 cm. Substrates were silicon wafer (10 mm × 15 mm) and glass (1″) and time deposition was chosen to obtain similar thickness of about 4 and 3 µm for PLD and sputtered films, respectively.

The chemical composition of Er^3+^-doped Ga-Ge-Sb-S targets and films were studied by scanning electron microscopy with an energy-dispersive X-ray analyzer (SEM-EDS, JSM 6400-OXFORD Link INCA) collecting the spectra over a roughly 0.01 mm^2^ area for each sample. Atomic percentage of each element (±0.5% and ±1% for the targets and the films, respectively) was extracted exploiting LLLK lines of Ga, Ge, Sb and S element working at 10 kV.

The morphology and the topography of pulsed laser deposited and sputtered films were analyzed by atomic force microscopy (AFM, Ntegra Prima, NT-MDT). The tapping mode imaging was used on area of 2 µm × 2 µm and 5 µm × 5 µm. The SEM technique was also applied to observe thin films morphology using a field emission gun SEM (JSM 6301 F).

Linear refractive indices of pulsed laser deposited, sputtered films and chalcogenide targets were obtained from the analysis VASE data measured using a rotating analyzer ellipsometer measuring in UV-Vis-NWIR (300–2500 nm) (J.A. Woollam Co., Inc., Lincoln, NE, USA). The VASE data were recorded at three angles of incidence (65°, 70° and 75° for thin films and 50°, 60° and 70° for targets). The resolution was set to 5 nm for the films considering the estimated thickness of chalcogenide films and 20 nm for Er^3+^-doped Ga-Ge-Sb-S targets. The Cody-Lorentz model^36,37^ was used to analyze VASE data. This model is appropriate for the description of amorphous chalcogenide optical functions^38^. The thickness of chalcogenide thin films was also determined by VASE data analysis.

The recorded Raman scattering spectra for Er^3+^-doped Ga-Ge-Sb-S bulk targets and films deposited by pulsed laser or sputtered by RF magnetron were collected by a LabRam HR800 spectrometer (Horiba Jobin-Yvon) equipped with a laser emitting at 785 nm (Toptica), an Olympus BX41 microscope with 100X objective and a confocal aperture. Raman spectroscopy is known to be a non-destructive technique: the laser power has been adjusted on the samples so that they are not damaged or modified under the excitation laser beam.

The optical and spectroscopic properties of the Er^3+^-doped films were obtained via fluorescence spectroscopy and lifetime measurements. An 808 and a 980 nm laser diode were used to excite the films and obtaining the emission of erbium at 1.55 µm by focusing the beam onto the sample. The luminescence was collected via a monochromator leading to an InGaAs detector. The same setup was used for investigating the NWIR emission bands at 980 nm and 1.55 µm and the MWIR emission band at ~2.7 µm, using a nitrogen-cooled InSb detector and exciting at 808 nm. The excitation beam was modulated at a 20 Hz frequency, and the emission intensity signal was processed through a lock-in amplifier to reduce environmental noise in the collection of fluorescence spectra.

Lifetime measurements were performed with a similar setup, using an oscilloscope, and using the modulation frequency signal from the diode drive as the trigger. The collected signal was averaged over more than 500 cycles to improve the signal-to-noise ratio.

The thin films samples were annealed below the glass transition temperature of corresponding glass target. The annealing was performed in a horizontal furnace: heating proceeded from room temperature up to 290 °C over a period of 4 hours and 30 minutes, then lingering at 290 °C for 2 hours, and cooling down naturally to room temperature overnight. The fluorescence and lifetimes of the annealed samples were then collected and compared to those obtained before the treatment.

## Conclusions

The development and characterization of optically active thin films from erbium-doped Ga-Ge-Sb-S glass opens the way for several applications in the infrared range. In this work, the results of two PVD methods, namely RF sputtering and PLD, for the fabrication of micrometer-thick films were compared. This study provides an extensive description of the effects of the different deposition parameters on the composition of the end product and on its optical and spectroscopic properties. It can be concluded that the most prominent factor for the optical properties of the films is the sulfur content, which will change the band-gap energy and the refractive index of the thin sulfide films doped with erbium ions. In turn, one can see that the latter is particularly affected by chamber pressure during deposition. The understanding of how the deposition parameters affect the composition, and therefore the properties of the resulting films, allows the fabrication of planar waveguides designed according to the specific needs of their intended application, such as sensing, signal manipulation, or the amplification of light. PLD and sputtering approaches have distinct advantages and disadvantages. PLD films have a more consistent composition-wise with respect to a possible variation in deposition parameters and their deposition rate is higher, making them a rather reliable deposition method. However, they strongly require an annealing process to homogenize the structure of the active ion centers and their optical properties appear more erratic, not showing well-define trends as a function of the deposition parameters and therefore less predictable. The properties of the sputtered films depend more strongly on the deposition parameters, making this method interesting for the fabrication of multi-layer or graded-index structures with a single target, and for tailoring the composition by controlling the deposition conditions. This deposition technique shows here its good suitability as rare earth doped chalcogenide films are necessary for applications without the need for post-deposition treatments.

## Data Availability

The datasets generated and analyzed during the current study are available from the corresponding author on reasonable request.
